# Chlamydiosis in British Garden Birds (2005–2011): Retrospective Diagnosis and *Chlamydia psittaci* Genotype Determination

**DOI:** 10.1007/s10393-014-0951-x

**Published:** 2014-06-20

**Authors:** K. M. Beckmann, N. Borel, A. M. Pocknell, M. P. Dagleish, K. Sachse, S. K. John, A. Pospischil, A. A. Cunningham, B. Lawson

**Affiliations:** 1 Institute of Zoology, Zoological Society of London, Regent’s Park, London, NW1 4RY UK; 2 Vetsuisse Faculty, Institute of Veterinary Pathology, University of Zurich, Winterthurerstrasse 268, 8057 Zurich, Switzerland; 3 Finn Pathologists, One Eyed Lane, Weybread, Diss, Norfolk, IP21 5TT UK; 4 Moredun Research Institute, Pentlands Science Park, Bush Loan, Penicuik, Edinburgh, EH26 0PZ Scotland, UK; 5 Friedrich-Loeffler-Institut (Federal Research Institute for Animal Health), Institute of Molecular Pathogenesis, Naumburger Str. 96a, 07743 Jena, Germany

**Keywords:** *Chlamydia psittaci*, chlamydiosis, collared dove *Streptopelia decaocto*, Order Passeriformes, passerine, wild bird

## Abstract

The significance of chlamydiosis as a cause of mortality in wild passerines (Order Passeriformes), and the role of these birds as a potential source of zoonotic *Chlamydia psittaci* infection, is unknown. We reviewed wild bird mortality incidents (2005–2011). Where species composition or post-mortem findings were indicative of chlamydiosis, we examined archived tissues for *C. psittaci* infection using PCR and ArrayTube Microarray assays. Twenty-one of 40 birds tested positive: 8 dunnocks (*Prunella modularis*), 7 great tits (*Parus major*), 3 blue tits (*Cyanistes caeruleus*), 2 collared doves (*Streptopelia decaocto*, Order Columbiformes), and 1 robin (*Erithacus rubecula*). *Chlamydia psittaci* genotype A was identified in all positive passerines and in a further three dunnocks and three robins diagnosed with chlamydiosis from a previous study. Two collared doves had genotype E. Ten of the 21 *C. psittaci*-positive birds identified in the current study had histological lesions consistent with chlamydiosis and co-localizing *Chlamydia* spp. antigens on immunohistochemistry. Our results indicate that chlamydiosis may be a more common disease of British passerines than was previously recognized. Wild passerines may be a source of *C. psittaci* zoonotic infection, and people should be advised to take appropriate hygiene precautions when handling bird feeders or wild birds.

**Electronic supplementary material:**

The online version of this article (doi:10.1007/s10393-014-0951-x) contains supplementary material, which is available to authorized users.

## Introduction

Chlamydiosis is a disease of birds and mammals, including people, caused by infection with the Gram-negative, intracellular bacterium, *Chlamydia* (*Chlamydophila*) *psittaci* (Family Chlamydiaceae, Order Chlamydiales) (Vanrompay et al. 1995; Andersen and Franson 2007). Birds are the primary hosts of *C. psittaci* (Andersen and Franson 2007), and a wide range of avian species is susceptible to infection (Kaleta and Taday 2003). Avian infections are frequently asymptomatic (Kaleta and Taday 2003) but can also cause a broad spectrum of disease (“avian chlamydiosis”) including respiratory, enteric, and ocular disease (Vanrompay et al. 1995; Andersen and Franson 2007). Gross lesions typically include air sacculitis, serositis, hepatomegaly, and splenomegaly (Vanrompay et al. 1995), and there is often concurrent infectious disease (Pennycott et al. 2009). Microscopic lesions are variable: splenic, hepatic, renal, and/or myocardial necrosis may be evident in acute cases; other findings can include splenic and/or hepatic histiocytosis, hepatic periportal inflammatory cell infiltrates, and biliary hyperplasia (Vanrompay et al. 1995).

Avian chlamydiosis has been diagnosed in a variety of wild bird species in Europe, particularly columbiforms (Order Columbiformes) such as collared doves (*Streptopelia decaocto*), feral pigeons (*Columbia livia*), and wood pigeons (*Columba palumbus*) (Bracewell and Bevan 1986; Magnino et al. 2009). Also, chlamydiosis has been diagnosed occasionally in passerines (Order Passeriformes) (Simpson and Bevan 1989; Holzinger-Umlauf et al. 1997; Pennycott et al. 2009). The first reported occurrence of the disease in passerines in Britain was in 1988, when robins (*Erithacus rubecula*), dunnocks (*Prunella modularis*) and Paridae (tit species) were affected in a garden in south-west England (Simpson and Bevan 1989). Subsequently, Pennycott et al. (2009) reported mortality of Fringillidae (finches), Paridae, and robins in a Scottish garden in 2008, in which trichomonosis was considered the primary cause of disease and death, but in which concurrent chlamydiosis was diagnosed in some of the birds examined. Colvile et al. (2012) described a further six incidents which affected Paridae and/or dunnocks and/or robins in England in 2009 (1 incident) and 2011 (5 incidents).

*Chlamydia psittaci* is currently classified into seven ompA genotypes, each of which appears to have a certain host predilection: genotype A (parrots), B (pigeons), C (ducks and geese), D (turkeys), E (pigeons, ducks and other species), F (parakeets), and E/B (ducks, turkeys and pigeons) (Vanrompay et al. 1997; Geens et al. 2005; Sachse et al. 2009). These data are derived mainly from studies of captive or farmed birds and feral pigeons: *C. psittaci* genotypes infecting wild passerines have rarely been determined (Kaleta and Taday 2003; Kalmar et al. 2013). In a recently proposed extension of the ompA typing scheme, subgroups of genotypes A (A-VS1, A-6BC and A-8455), E/B (EB-E30, EB-859 and EB-KKCP), and D (D-NJ1 and D-9N) were described, and six further avian genotypes were identified [in corvids, parrots, an oriental stork (*Ciconia boyciana*), and a brown skua (*Stercorarius antarcticus lonnbergi*)] (Sachse et al. 2008).

While pigeons and doves appear to be the major wild bird reservoir of *C. psittaci* across Europe (Bracewell and Bevan 1986; Magnino et al. 2009), variable and potentially high prevalences of *C. psittaci* infection have been demonstrated in some wild passerine populations. For example, in Germany, 215 of 399 (54%) clinically healthy tits [including 30 of 43 (70%) blue tits (*Cyanistes* (*Parus*) *caeruleus*), 169 of 318 (53%) great tits (*Parus major*) and 12 of 32 (38%) marsh tits (*Poecile* (*Parus*) *palustris*)] were found to be *Chlamydia* sp. positive from cloacal and pharyngeal swabs using immunofluorescent antibody testing (Holzinger-Umlauf et al. 1997). Olsen et al. (1998) detected *C. psittaci* in 9 of 219 (3%) passerines sampled in Sweden (using PCR on fecal samples), including 2 of 29 (7%) robins and 1 of 21 (5%) great tits. Observation of sick birds was not reported; therefore, it seems likely that birds sampled in this study were apparently healthy. Others have failed to detect *C. psittaci* infection in passerines: Zweifel et al. (2009) from 527 passerines [including 211 chaffinches (*Fringilla coelebs*), 47 great tits and 12 robins] sampled in Switzerland (by PCR on cloacal swabs), and Prukner-Radovćic et al. (2005) from 53 passerines (including 15 robins) sampled in Croatia (by ELISA on cloacal swabs). The prevalence of *C. psittaci* infection in wild passerines in Britain is unknown.

*Chlamydia psittaci* infection causes a range of symptoms in human beings (in which the disease is termed “psittacosis”), ranging from asymptomatic infection or mild, flu-like illness to severe respiratory disease that, in rare cases, can be fatal (Smith et al. 2011; Rehn et al. 2013). Human cases have most often been attributed not only to direct or indirect contact with infected captive psittacine birds (Palmer 1982; Wreghitt and Taylor 1988; Smith et al. 2011), but also to contact with poultry (particularly ducks) (Palmer 1982; Gaede et al. 2008; Laroucau et al. 2009) and racing and feral pigeons (Haag-Wackernagel and Moch 2004; Harkinezhad et al. 2009; Magnino et al. 2009). The origins of human psittacosis cases, however, are often undetermined [e.g., Health Protection Agency (HPA), and Department for Environment, Food & Rural Affairs (Defra) 2012]. Other wild bird species have been implicated in some psittacosis outbreaks (Williams et al. 1998; Telfer et al. 2005; Herrmann et al. 2006; Rehn et al. 2013), including wild passerines, which were the suspected source of an outbreak that affected at least 25 people in southern Sweden in early 2013 (Rehn et al. 2013).

Wild bird carcasses tend not to be tested for *C. psittaci* infection routinely due to financial constraints (molecular tests are required to obtain a diagnosis) (Pennycott et al. 2009); therefore, the prevalence of chlamydiosis in British passerines has been under-investigated (Colvile et al. 2012). Here, we conducted a retrospective survey of selected garden bird carcasses submitted by members of the public across England and Wales in order to investigate the significance of chlamydiosis as a cause of disease in these species. We use the term “chlamydiosis” to describe cases in which *C. psittaci* infection was detected in birds which had gross, histological, and immunohistochemical findings consistent with the disease. We conducted *C. psittaci* genotyping of positive cases in order to further our understanding of the epidemiology of the infection in British garden birds.

## Methods

### Wild Bird Cases

Reports of sick and dead wild birds were received from the general public through a national disease surveillance network established as part of the Garden Bird Health *initiative* (GBH*i*) (Robinson et al. 2010). Morbidity and mortality incidents were reported either on an ad hoc basis or through a systematic volunteer scheme (Robinson et al. 2010). A detailed description of each incident was obtained, including the species and number of birds affected, date when sick and/or dead birds were first observed, location, and clinical signs. If available, carcasses suitable for post-mortem examination (PME) were submitted.

On receipt, carcasses were either refrigerated at 4°C and examined within 48 h, or frozen at −20°C and examined at a later date. PMEs followed a standardized protocol, as described by Lawson et al. (2011). Birds were assigned to the age classes “Nestling,” “Juvenile,” (fully fledged and independent from nest) or “Adult” (any individual beyond its post-juvenile molt), and sex was determined, where possible, on the basis of plumage characteristics or gonad inspection. Carcasses were weighed, and body condition was subjectively assessed (as “Emaciated,” “Thin,” “Normal,” or “Fat”) on the basis of subcutaneous fat deposits and pectoral muscle condition. Samples (liver, small-intestinal content, and tissues with macroscopic lesions) were routinely submitted for microbiological examination using a standardized protocol (Lawson et al. 2011). A saline mount preparation of small-intestinal contents was examined microscopically for parasites. A standard range of tissues from each case was frozen at −20°C pending further testing and, where the state of carcass preservation permitted, tissue samples were fixed in neutral-buffered 10% formalin pending histological examination. Tissues were submitted for further tests (in addition to those described below) as indicated by the macroscopic findings, including culture and PCR to detect *Trichomonas* sp. infection (Robinson et al. 2010), and histopathology and PCR to detect avipoxvirus infection (Lawson et al. 2012).

Cases were selected for *C. psittaci* testing from an archive of 1,578 passerine and columbiform carcasses received at the Institute of Zoology from across England and Wales, 2005–2011, on the basis of either (1) having gross lesions consistent with previously reported chlamydiosis incidents (hepatomegaly and/or splenomegaly and/or serositis), or (2) having been from a mortality incident in which the species assemblage of sick and dead birds was consistent with previously reported passerine chlamydiosis incidents (involvement of robins and/or Paridae and/or dunnocks). In addition, tissues from six passerine carcasses in which chlamydiosis had already been diagnosed (Colvile et al. 2012) were submitted for molecular *C. psittaci* testing.

### Molecular Detection of *C. psittaci* Infection

DNA was extracted from frozen/thawed liver, or from pooled liver and spleen where both archived tissues were available, using a Biosprint 15 DNA Blood Kit (Qiagen Ltd., Manchester, M15 6SH, UK) according to the manufacturer’s instructions. The purified DNA was stored at 4°C, until the molecular analyses were performed.

All samples were examined by real-time PCR with primers specific for the 23S rRNA gene (Family Chlamydiaceae) using an ABI 7500 thermocycler (Applied Biosystems, Foster City, California, USA) following methods described by Ehricht et al. (2006) and Zweifel et al. (2009) which had a detection limit of 1 inclusion-forming unit (ifu) (Ehricht et al. 2006). A positive control (*C. abortus* DNA) and a negative control (reaction mix with molecular biology grade water) were included in each PCR run. Each sample was tested in duplicate. When both Ct-values were <38, a sample was considered as positive (Zweifel et al. 2009). Samples for which one or both duplicates gave Ct-values of >38 were considered as questionably positive. If amplification was absent in both duplicates, the sample was interpreted as negative, and no further molecular tests were performed. Positive and questionably positive samples were further examined using each of the following three tests:

1. A *Chlamydia* species-specific 23S ArrayTube (AT) Microarray assay (Alere Chip Technologies GmbH, Jena, Germany) as described by Borel et al. (2008), which had a detection limit of 1 ifu (Ehricht et al. 2006).
2. A *C. psittaci* ompA real-time PCR, which had a detection limit of 2 ifu, as described by Pantchev et al. (2009). Each sample was tested in duplicate with positive (*C. psittaci* DNA) and negative (molecular grade water) controls included. A sample was considered as positive when the average Ct-value was <36 (Pantchev et al. 2009), and as questionable positive when the average Ct-value was >36.
3. A *C. psittaci* genotyping assay, as described by Sachse et al. (2008). In the case of weak signals where the ompA genotype could not be accurately identified by the software, the assignment was done manually based on the closest match: these cases were termed “weak positive.” The lowest amount of DNA required for correct typing was equivalent to 2 ifu (Sachse et al. 2009).

Samples were considered positive for *C. psittaci* if they were positive (including—for the *C. psittaci* genotyping assay—“weak positive”) on at least one of these three further tests.

### Histology and Immunohistochemistry

In *C. psittaci*-positive cases for which formalin-fixed tissues were available, the significance of the infection was investigated using histopathological examination and immunohistochemistry.

Formalin-fixed tissues were prepared for histopathological examination using routine methods (Bancroft 2008), and 5-µm-thick sections were examined using various stains including H&E, Ziehl-Neelsen, Giemsa, Periodic Acid-Schiff, and Gram-Twort.

*Chlamydia* spp.-specific immunohistochemistry, using anti-chlamydial lipopolysaccharide antibody (mouse IgG_1_, clone 13/4; Santa Cruz Biotechnology Inc., California, USA), was performed on paraffin-embedded, formalin-fixed tissue sections following the methodology described by Buxton et al. (1996).

A diagnosis of chlamydiosis was made for *C. psittaci-*positive cases which had co-localization of *Chlamydia* spp.-specific immunolabeling with histological lesions consistent with the disease [such as splenic, hepatic, renal and/or myocardial necrosis, splenic and/or hepatic histiocytosis, hepatic periportal inflammatory cell infiltrates, and biliary hyperplasia (Vanrompay et al. 1995)].

## Results

### Wild Bird Cases

Tissues from 40 birds (from 38 mortality incidents) in the case archive fulfilled our selection criteria and were tested for *C. psittaci* infection using molecular methods. These comprised 35 passerines (from 33 mortality incidents) and 5 columbiforms (from a further 5 mortality incidents) (Table 1).

**Table 1:** Number of birds of each species submitted for molecular testing for *C. psittaci* infection and summary of results

Taxonomic group	No. cases tested and results		
	Positive	Negative	Total
Order Passeriformes			
Family Paridae			
Great tit (*Parus major*)	7	5	12
Blue tit (*Cyanistes caeruleus*)	3	1	4
Family Prunellidae			
Dunnock (*Prunella modularis*)	8	0	8
Family Turdidae			
Robin (*Erithacus rubecula*)	1	3	4
Family Corvidae			
Rook (*Corvus frugilegus*)	0	2	2
Jackdaw (*Corvus monedula*)	0	2	2
Family Fringillidae			
Chaffinch *(Fringilla coelebs*)	0	1	1
Family Troglodytidae			
Wren (*Troglodytes troglodytes*)	0	1	1
Family Motacillidae			
Pied wagtail (*Motacilla alba*)	0	1	1
Order Columbiformes			
Family Columbidae			
Collared dove (*Streptopelia decaocto*)	2	1	3
Feral pigeon (*Columba livia*)	0	2	2
Total	21	19	40

### Molecular Detection of *C. psittaci* Infection

Tissues from 21 of the 40 selected cases tested positive for *C. psittaci* DNA: all of 8 dunnocks, 7 (of 12) great tits, 3 (of 4) blue tits, 2 (of 3) collared doves, and 1 (of 4) robins (Table 1 and Supplementary Table 1). For the positive cases, the results of each of the molecular tests are presented in Table 2. The 21 positive cases had been submitted from 20 separate mortality incidents, the details of which are presented in Table 3. Each of 4 corvids, 2 feral pigeons, 1 wren (*Troglodytes troglodytes*), 1 chaffinch, and 1 pied wagtail (*Motacilla alba*) tested were negative.

**Table 2:** Results of PCR, ArrayTube Microarray, and genotyping assays in *C. psittaci*-positive birds

Case no.	Species	23S rtPCR for *Chlamydiaceae* ^b^		23S ArrayTube Microarray^d^	*C. psittaci* ompA rtPCR^e^		*C. psittaci* genotyping assay^f^
		Ct-value	Result		Ct-value	Result	
1	Blue tit	39.6	Ques	Neg	38.6	Ques	Genotype A-6BC
2	Dunnock	17.2	Pos	*C. psittaci*	18.0	Pos	Genotype A-VS1
3	Dunnock	41.3	Ques	Neg	38.6	Ques	Weak positive
4	Dunnock	18.8	Pos	*C. psittaci*	20.4	Pos	Genotype A-VS1
5	Great tit	26.3	Pos	*C. psittaci*	27.5	Pos	Genotype A-VS1
6	Great tit	38.9^c^	Ques	*C. psittaci*	38.8	Ques	Weak positive
7	Great tit	37.1	Pos	*C. psittaci*	38.2	Ques	Genotype A-VS1
8	Great tit	26.0	Pos	*C. psittaci*	27.0	Pos	Genotype A-VS1
9	Dunnock	21.9	Pos	*C. psittaci*	22.7	Pos	Genotype A-VS1
10	Collared dove	15.2	Pos	*C. psittaci*	20.1	Pos	Genotype E
11	Dunnock	19.8	Pos	*C. psittaci*	23.9	Pos	Genotype A-VS1
12	Dunnock	15.9	Pos	*C. psittaci*	16.9	Pos	Genotype A-VS1
13	Robin	40.5^c^	Ques	Neg	29.6	Pos	Neg
14	Blue tit	43.7^c^	Ques	*C. psittaci*	40.0	Ques	Neg
15	Collared dove	13.9	Pos	*C. psittaci*	17.7	Pos	Genotype E
16	Great tit	26.5^c^	Ques	*C. psittaci*	30.6	Pos	Genotype A-6BC
17	Dunnock	14.6	Pos	*C. psittaci*	19.2	Pos	Genotype A-6BC
18	Great tit	25.9^c^	Ques	*C. psittaci*	30.0	Pos	Genotype A-6BC
19	Dunnock	15.9^c^	Ques	*C. psittaci*	20.9	Pos	Genotype A-VS1
20	Great tit	19.0	Pos	*C. psittaci*	23.4	Pos	Genotype A-VS1
21	Blue tit	34.3	Pos	Neg	39.5	Ques	Genotype A-VS1
Six further cases described in a previous study^a^							
	Robin	16.7	Pos	*C. psittaci*	18.1	Pos	Genotype A-VS1
	Robin	15.6	Pos	*C. psittaci*	19.9	Pos	Genotype A-VS1
	Dunnock	19.1	Pos	*C. psittaci*	23.2	Pos	Genotype A-VS1
	Robin	13.0	Pos	*C. psittaci*	17.7	Pos	Genotype A-VS1
	Dunnock	23.8^c^	Ques	*C. psittaci*	27.9	Pos	Genotype A-VS1
	Dunnock	11.8	Pos	*C. psittaci*	16.5	Pos	Genotype A-VS1

Samples were considered positive for *C. psittaci* if they were (1) positive or questionably positive on 23S PCR, and (2) positive (including, for the *C. psittaci* genotyping assay, “weak positive”) on at least one of the subsequent molecular tests.

*Pos* positive, *Ques* questionable positive, *Neg* negative.

^a^Six additional chlamydiosis cases reported by Colvile et al. (2012) also submitted for molecular testing.

^b^23S rtPCR as described by Ehricht et al. (2006) and Zweifel et al. (2009). Ct-value averaged from two duplicate samples, cut-off value 38.0.

^c^Only one Ct-value was determined.

^d^23S ArrayTube Microarray assay as described by Borel et al. (2008).

^e^OmpA rtPCR as described by Pantchev et al. (2009). Ct-value averaged from at least two duplicate samples, cut-off value 36.0.

^f^ *C. psittaci* genotyping assay as described by Sachse et al. (2008). In the case of weak signals where the ompA genotype could not be accurately identified by the software, the assignment was done manually based on the closest match: these cases were termed “weak positive.”

**Table 3:** Details of mortality incidents and gross post-mortem examination findings in *C. psittaci*-positive birds

Case no.	Species and signalment	Details of mortality incident			Body condition, [bodyweight (g)] and gross findings on post-mortem examination
		Date and location	Species affected: no. birds found dead (no. seen sick) (*and total no. affected individuals*)	Clinical signs (if sick birds were observed) and/or perceived cause of death (reported by members of the public)	
1	Blue tit Adult	Oct 2005 Wiltshire, England	Blue tit 1 (0)	None reported	Normal (11.1) Suspected hepatomegaly
2	Dunnock Adult male	Jan–Feb 2006 East Sussex, England	Dunnock 2 (1) (*2 individuals*)	One individual was fluffed up prior to death	Emaciated (17.4) Suspected splenomegaly
3	Dunnock Adult	Sep 2006–Jan 2007 Staffordshire, England	Dunnock 1 (1) (*1 individual*)	Dunnock was fluffed up and lethargic prior to death	Thin (17.0) Hepatomegaly. Necrotic ingluvitis
			Greenfinch 6 (some)	Some greenfinches were fluffed up and unable to fly	
			Chaffinch 14 (some)	None reported	
			House sparrow 2 (0)	None reported	
4	Dunnock Adult female	Feb 2007 Northamptonshire, England	Dunnock 1 (0)	Suspected window strike	Emaciated (13.4) Suspected splenomegaly
5	Great tit Adult female	Apr 2007 Wrexham, Wales	Great tit 1 (0)	None reported	Thin (14.1) Penetrating wound, rib fractures and fibrinous serositis
6	Great tit Adult	Sep–Oct 2007 East Sussex, England	Great tit 1 (0) Greenfinch 0 (1)	Great tit was predated by a cat Greenfinch was fluffed up and lethargic	Normal (20.5) Splenomegaly. Pedunculated skin lesions on wing. Puncture wound
7	Great tit Adult	Jul–Sep 2007 Surrey, England	Great tit 3 (3) (*≥4* *individuals*) Blue tit 5 (0)	Multiple individuals had skin growths, particularly on face and wing. Two of the dead great tits were euthanized	Normal (17.6) Splenomegaly. Facial skin lesions. Hemorrhage (euthanasia)
8	Great tit Adult male	Jul–Oct 2007 East Sussex, England	Great tit 2 (3) (*3 individuals*)	Two great tits were lethargic and one other was observed to have a skin growth on wing	Normal (16.9) Splenomegaly. Suspected hepatomegaly. Fibrinous serositis. Hemorrhagic, inflamed neck lesion
			Dunnock 1 (1) (*1 individual*)	Dunnock was fluffed up before death	
9	Dunnock Adult	From the same mortality incident as Case 8 (see above)			Emaciated (15.9) Hepatomegaly and splenomegaly
10	Collared dove Adult female	Sep 2008 Essex, England	Collared dove 1 (1) (*1 individual*)	Found sick following cat predation and later died	Emaciated (108.5) Serositis, air sacculitis and pericarditis. Ingluvitis. Hepatomegaly
11	Dunnock Adult male	Nov 2008–Jan 2009 Powys, Wales	Dunnock 2 (2) (*2 individuals*) Robin 1 (1) (*1 individual*)	Dunnocks and robin were fluffed up and lethargic before death	Emaciated (15.7) Anorexia
			Greenfinch 0 (1)	None reported	
12	Dunnock Adult male	Feb 2009 West Sussex, England	Blue tit 1 (0)	Blue tit was a possible window strike	Thin (19.2) Fractures with no associated hemorrhage
			Dunnock 3 (0)	One dunnock was a possible window strike	
			Great tit 3 (0)	One great tit had avian pox (confirmed post-mortem)	
			Robin 2 (0)	None reported	
			Pheasant 1 (0)	None reported	
13	Robin Nestling	Apr 2009 Surrey, England	Robin 3 (0)	All of a clutch of 3 nestlings found dead	Thin (11.0) Hepatic congestion
14	Blue tit Nestling	May 2009 Staffordshire, England	Blue tit 6 (0)	Six of a clutch of 7 nestlings died	Thin (5.3) Suspected hepatomegaly. Anorexia
15	Collared dove Juvenile	Jun 2009 Tyne and Wear, England	Collared dove 1 (1) (*1 individual*)	Fledgling, seen lethargic before death	Emaciated (104)
					Hepatomegaly, splenomegaly and serositis
16	Great tit Adult female	Feb 2010 Wiltshire, England	Great tit 1 (1) (*1 individual*)	Lethargic prior to death, with skin lesion on head	Thin (15.5) Large skin lesion on head. Suspected splenomegaly
17	Dunnock Adult male	Mar 2010 Kent, England	Dunnock 1 (1) (*1 individual*)	Dunnock was fluffed up and lethargic then predated by a cat	Thin (17.0) Wound, fracture and hemorrhage. Splenomegaly and suspected hepatomegaly. Numerous intestinal helminths
			Blue tit 0 (1)	Blue tit was observed to be “sick”	
18	Great tit Adult female	Feb–Apr 2010 Surrey, England	Great tit 1 (5) (*5 individuals)*	Multiple great tits had fleshy skin growths, one died	Thin (13.7) Multiple skin lesions. Suspected splenomegaly. Numerous lice
19	Dunnock Adult male	Apr 2010 Hampshire, England	Dunnock 1 (1) (*1 individual*)	Fluffed up and lethargic, euthanized	Thin (17.0) Wounds, fractures and hemorrhage (euthanasia). Hepatomegaly and suspected splenomegaly
20	Great tit Adult female	Oct 2010 Surrey, England	Great tit 1 (0)	Skin lumps, cat predation	Normal (19.0) Multiple skin lesions. Fracture and hemorrhage. Splenomegaly
21	Blue tit Adult male	Mar–Apr 2011 Worcestershire, England	Blue tit 6 (≥9) Great tit 0 (≥2)	Sick blue tits and great tits were lethargic and some appeared to have dyspnea. Some appeared to have epiphora and/or blepharitis and/or blepharospasm. At least two sick blue tits were euthanized and some died	Thin (8.16) Pulmonary congestion. Anorexia

Nine *C. psittaci*-positive birds were from eight incidents of multi-species passerine mortality; eight positive birds were from incidents in which only a single bird had been observed to be sick or found dead; and four positive birds were from sites of multiple mortality where a single species had been affected [including two nestlings—a robin (Case 13) and a blue tit (Case 14)—from failed nests] (Table 3). Positive cases had either been observed with non-specific clinical signs (fluffed up plumage and/or lethargy) prior to death (11 cases), had been found dead (4 cases), had suffered trauma (including predation) (at least 6 cases), or had been euthanized for welfare reasons (2 cases). One positive blue tit (Case 21) had been submitted from an incident in which it, and other blue tits and great tits, had been observed with apparent dyspnea and ocular disease. Two positive collared doves were from separate incidents where no other sick or dead birds were observed; there was no report of columbiform morbidity or mortality at any of the positive passerine incidents (Table 3).

**Figure 1:**
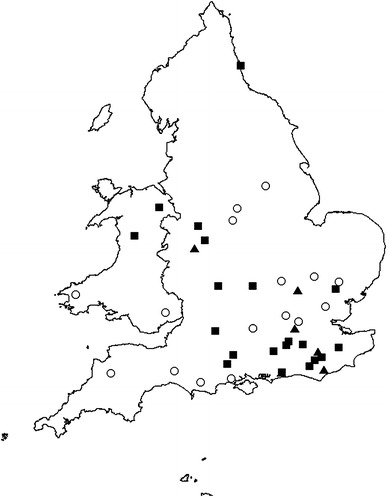
Geographical distribution of garden birds tested for *C. psittaci* (2005–2011). *Closed squares* represent sites from which *C. psittaci-*positive birds were submitted; *closed triangles* represent sites from which six additional positive birds (described by Colvile et al. 2012) were submitted; and *open circles* represent sites from which birds negative for *C. psittaci* were submitted.

Eighteen of the 21 *C. psittaci*-positive birds were adults, comprising 7 males (4 great tits, 2 dunnocks, and 1 blue tit), 6 females (4 great tits, 1 dunnock, and 1 collared dove), and 5 birds of undetermined sex (2 great tits, 2 dunnocks and 1 blue tit) (Table 3). The remaining positive birds were nestlings (see above) and a juvenile collared dove (Table 3). Positive cases had been submitted in each year of the study: 1 (of 1 tested) was from 2005, 1 (2) from 2006, 7 (12) from 2007, 1 (4) from 2008, 5 (9) from 2009, 5 (8) from 2010, and 1 (4) was from 2011. The positive birds had been found dead in all seasonal quarters of the year: 7 had been found in January-March; 7 in April–June; 2 in July–September; and 5 in October–December. Figure 1 shows the locations of positive and negative cases.

The *C. psittaci* genotype involved was determined for 17 of the 21 positive birds (Table 2). Genotype A was present in all 15 passerine cases for which the genotype was determined (7 dunnocks, 6 great tits and 2 blue tits) and was subtyped as genotype A-VS1 in 11 cases (6 dunnocks, 4 great tits and 1 blue tit) and as genotype A-6BC in 4 cases (2 great tits, 1 dunnock, and 1 blue tit). A further 3 dunnocks and 3 robins confirmed to have chlamydiosis in a previous study (Colvile et al. 2012) were also found to have been infected with genotype A-VS1. The two positive collared doves examined were infected with *C. psittaci* genotype E.

### Pathological Examination

**Figure 2:**
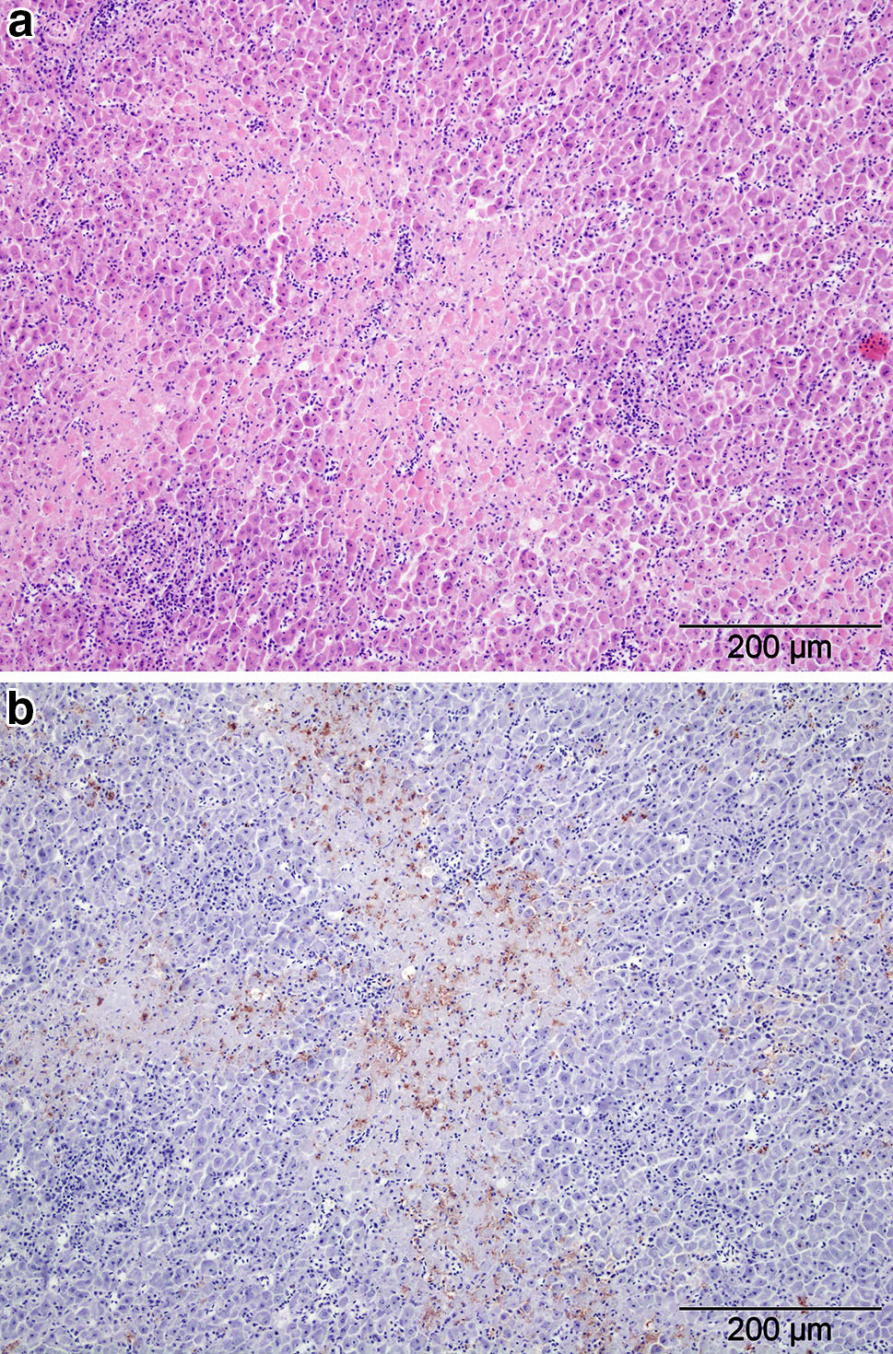
Liver of a dunnock (*Prunella modularis*) (Case 9), showing **a** multiple random foci of coagulative hepatocellular necrosis on H&E stain and **b** semi-serial section of liver subjected to immunohistochemistry (IHC) for *Chlamydia* spp. bacteria specific LPS: note positive labeling (red/brown pigment) in the cytoplasm of many of the necrotic hepatocytes (central area with pale blue poorly demarcated cells) and also some viable hepatocytes (IHC with haematoxylin counter-stain).

Of the 21 *C. psittaci*-positive birds, the state of carcass preservation in six birds precluded histopathological or immunohistochemical evaluation (Supplementary Table 2). Of the 15 birds for which tissues were examined microscopically, the significance of *C. psittaci* infection was unclear in five (Supplementary Table 2), but chlamydiosis was diagnosed by histological and immunohistochemical examination in 10 (Table 4 and Fig 2): 5 dunnocks, 3 great tits, and 2 collared doves, from 9 separate mortality incidents. Of the chlamydiosis cases, body condition was “emaciated” in 6 cases, “thin” in 3 cases, and “normal” in 1 case; splenomegaly was suspected/confirmed in 7 cases, hepatomegaly was suspected/confirmed in 5 cases, and serositis was present in 4 cases (Table 4).

**Table 4:** Pathological findings from wild birds with chlamydiosis

Case no.	Species and signalment	Results of microbiological examination and additional tests	Histopathological findings	Immunohistochemical labeling for *Chlamydia* sp. specific antigens	Diagnoses
2	Dunnock (Adult male)	Liver and small intestine (SI): *Escherichia coli* 1. Spleen: no growth	Fibrinous to histiocytic hepatitis with fibrinous thrombosis of the hepatic veins. Fibrinonecrotic, focally extensive splenitis. Fibrinous pneumonia. Histiocytic, focal, mild epicarditis. Proventriculus and gizzard: histiocytic serositis. Giemsa-positive granules in Kupffer cells of the liver, with similar material in some dissociated cells (macrophages or autolyzed hepatocytes), possibly representative of Chlamydial inclusions (or conventional bacteria, such as the *E. coli* 1 isolated from the tissue). Ziehl-Neelsen (ZN) stain negative for acid-fast agents or inclusions	Intense positive immunolabeling in the heart (endo- and epicardium plus interstitial cells) and serosal surface of the trachea. Foci of positive labeling in the meninges, proventriculus and gizzard. Within the lung, spleen and liver, positive labeling in the cytoplasm of macrophage-like cells (possibly Kupffer cells in the liver)	Chlamydiosis; possible additional bacterial infection
4	Dunnock (Adult female)	Liver: mixed growth, predominantly *E. coli* 1. SI: *E. coli* 1 and *Enterococcus* sp.	Fibrinous to histiocytic hepatitis, with fibrinous thrombosis of hepatic veins. Fibrinous pneumonia. Marked atrophy of epicardial adipose tissue and pectoral muscle. Giemsa-positive, granular to linear material in Kupffer cells of the liver, possibly representative of Chlamydial inclusions (or conventional bacteria, such as the *E. coli* 1 isolated from the tissue; interpretation hindered by autolysis). ZN stain negative for acid-fast agents or inclusions	Positive immunolabeling in the heart (interstitium of the left ventricular wall, right ventricular wall cardio-myocytes, and epicardium), liver (macrophages, hepatocytes and white blood cells) and lung	Chlamydiosis; possible coli-septicemia; possible window strike
5	Great tit (Adult female)	Liver: confluent mixed growth. *E. coli* 1, *Moellerella wisconsensis* & *Enterococcus* spp.. Lung: mixed growth predominance *Serratia fonticola* & *M. wisconsensis*. Coelomic cavity: few colonies *E. coli* 1, *M. wisconsensis* & *Enterococcus* spp.	Fibrinous perihepatitis and striking cellular infiltrate of portal tracts throughout the liver parenchyma (interpretation hindered by autolysis). Multifocal, acute pulmonary edema. Moderate to marked atrophy of epicardial adipose tissue. ZN and Giemsa stain reveal no Chlamydial inclusions	Positive labeling in the heart (mainly associated with blood vessels), liver (hepatocytes and possibly Kupffer cells), kidney (interstitial tissue) and keel	Chlamydiosis; trauma (possible predation); possible additional (bacterial/viral) infection
8	Great tit (Adult male)	Lung, skin lesion and coelom: *E. coli* 1 & *Enterococcus* spp.	Vascular endothelial hypertrophy within heart and spleen, with intralesional Gram-negative organisms. Fibrinogranulomatous to mixed cellular, locally extensive, epicarditis, with intralesional Gram-negative organisms. Granulomatous to hemorrhagic, extensive dermatitis, possibly associated with an unidentified mite. Fibrinonecrotizing, focal, acute hepatitis. Fibrinonecrotizing splenitis. Mild pectoral muscle atrophy. Gram-Twort stain shows intra-endothelial organisms as Gram-negative coccobacilli or short rods and shows similar organisms in some epicardial macrophages. ZN and Giemsa stains show no evidence of Chlamydial inclusions	Positive labeling in the heart (epicardium and heart base), spleen (white blood cells), lung (parenchyma and white blood cells), liver (cell-associated, probably macrophages), and skin (inflammatory cells)	Chlamydiosis; possible other bacterial infection
9	Dunnock (Adult)	Liver: Mixed growth. *E. coli* 1 and *Providencia stuartii*. SI and bursa of Fabricus: *E. coli* 1	Fibrinonecrotic, marked hepatitis with multifocal probable fibrinous thrombosis and with intralesional bacterial rods. Fibrinonecrotic splenitis. Fibrinous pneumonia with intrahistiocytic bacterial rods. Focal epicarditis. Giemsa stain shows moderate numbers of bacterial rods in blood vessels in all tissues, within pulmonary macrophages and within some of the fibrinous lesions in the liver and spleen. ZN stain negative for acid-fast agents	Positive labeling in the liver (cell-associated and extracellular labeling in sinusoids and blood vessels), spleen (sub-capsule area), heart (interstitial tissue and myocardium), lung (white blood cells and pleura), and trachea (serosal surface)	Chlamydiosis; possible other bacterial infection
10	Collared dove (Adult female)	Liver, SI, crop, pericardium and lung: mixed, *E. coli* 1 and *Enterococcus* spp.. Crop: also *Candida tropicalis* & *C. albicans* 1. Crop tissue negative for *Trichomonas* sp. on culture and PCR	Severe candidiasis (crop mucosa markedly thickened, containing massive numbers of *Candida* sp. spores and pseudohyphae) and secondary bacterial infection (consistent with *E. coli* 1 infection isolated on culture). Marked, necrotic pericarditis. Liver, gizzard and small intestine: serocoelomitis. Scattered cells within the spleen appear to contain large, basophilic, cytoplasmic inclusions (autolysis hinders interpretation)	Positive specific labeling in the liver, spleen, serosal surface of the small intestine and individual cells (presumed macrophages) in the lung	Chlamydiosis; cat predation; candidiasis
11	Dunnock (Adult male)	SI content: *Campylobacter* sp.. Liver and lung: no growth	Granulocytic enteritis associated with luminal and encysted trematode life stages (consistent with schistosomes but autolysis hinders interpretation). Fibrinonecrotic hepatitis. Focal epicarditis. Mild pulmonary edema (probably agonal). Sarcocystosis of the pectoral muscle. Severe atrophy of epicardial adipose tissue. ZN stain shows no acid-fast agents or inclusions. Giemsa stain faintly highlights Sarcocysts in the pectoral muscle and highlights scanty punctate material in the foci of hepatic necrosis (nuclear dust, or less likely, bacteria or Chlamydial inclusions)	Positive labeling in the lung (diffuse, in cells resembling macrophages and within blood vessels), trachea (serosal surface and intramuscular), pectoral muscle, liver (diffuse, and some associated with bile duct epithelium), proventriculus and gizzard (interstitium and mucosa), heart (cell-associated in interstitium, and myocardium), spleen, and intestines (serosal surface and mucosa)	Chlamydiosis; possible other septicemia; parasitic enteritis; sarcocystosis (probably incidental); intestinal *Campylobacter* sp. infection (probably incidental)
15	Collared dove (Juvenile)	Liver: moderate pure growth *E. coli* 1. SI: confluent nearly pure *E. coli* 1. Crop: *Trichomonas* sp. isolated in Bushby’s medium (subclinical infection). Circovirus-specific PCR on necrotic coelomic tissue negative^a^	Fibrinogranulomatous, extensive serositis with intralesional Gram-negative bacteria and some plant material (possible artefactual transfer, or alimentary tract rupture). Fibrinonecrotic splenitis with intralesional Gram-negative bacteria. Diffuse, marked atrophy of adipose tissue. Giemsa stain highlights bacteria in the coelomic exudate but shows no inclusions. A Periodic Acid-Schiff (PAS) preparation highlights plant matter in the exudate on the stomach and intestine but shows no fungal agents. A Gram-Twort highlights the Gram-negative bacteria as coccobacilli to short rods. ZN stain shows no acid-fast agents or inclusions	Positive labeling in the heart (predominantly cell-associated but also extracellular, often perivascular), spleen (capsule and parenchyma), crop (serosal surface), proventriculus and gizzard (serosal surfaces and intramuscular), intestine (within inflammatory cells on the serosal surface), lung (within macrophages in alveoli and interstitium), and kidney (interstitium)	Chlamydiosis; possible other Gram-negative bacterial infection; possible alimentary tract rupture; subclinical *Trichomonas* sp. infection
16	Great tit (Adult female)	Liver: Light nearly pure growth of *Serratia ficaria*. Small intestine and lung: no growth. Skin lesion: avipox PCR positive	Generalized vascular endothelial hypertrophy in most tissues, with intralesional Gram-negative, PAS-positive organisms. Acute, fibrinous pneumonia with atelectasis. Fibrinonecrotic, extensive hepatitis. Fibrinonecrotic, disseminated, acute or subacute splenitis. Proliferative, multifocally necrotizing, extensive, severe dermatitis with numerous intracytoplasmic inclusion bodies (pathognomonic for avian poxvirus infection) and with minor surface infection by bacterial cocci and mixed fungi. Apparent mild hemoparasitism (compatible with leucocytozoonosis, but other hemoprotozoa could be indistinguishable on histology). ZN and Giemsa stains show no inclusions. Gram-Twort stain shows many of the endothelial bodies as Gram-negative coccoid or short bacillary structures; PAS stain highlights most of the same structures in intense magenta	Positive immunolabeling in the liver (cell-associated, primarily perivascular) and head lesions (inflammatory cells, primarily macrophages). (Brain, trachea, heart, pectoral muscle, lung, esophagus, spleen, proventriculus and gizzard and large intestine devoid of immunolabeling)	Chlamydiosis; avian pox disease with secondary mixed infection; hemoparasitism (significance unclear)
17	Dunnock (Adult male)	Liver, SI and peritoneum: pure isolate *E.coli* 1.	Fibrinonecrotic splenitis with intralesional, coccoid to coccobacillary, Gram-negative bacteria. Pulmonary congestion, edema and atelectasis. Generalized perivascular cellular infiltrates (interpretation hindered by autolysis). Sarcocystosis of the pectoral muscle with no evidence of myositis. ZN and Giemsa stains reveal no Chlamydial inclusions. A Gram-Twort stain shows the splenic bacteria to be Gram-negative, and apparently coccoid to coccobacillary	Positive labeling in the brain (cell-associated and possibly extracellular), trachea (muscle), lung (diffuse, cell-associated and extracellular), heart (diffuse, myocardium), crop (serosa), proventriculus (white blood cells within mucosa and lamina propria), gizzard (mucosa), liver (diffuse, cell-associated and extracellular), spleen (sub-capsular region, associated with white blood cells), large and small intestines, kidney (cell-associated), testis (cell-associated, interstitium), pectoral muscle (myofibrils)	Chlamydiosis; possible other bacterial sepsis; cat predation; heavy intestinal helminth burden; sarcocytosis (presumed incidental infection)

^a^Circovirus PCR performed by Biobest Laboratories Ltd., Penicuik, Scotland, EH26 0PY, UK.

There was concurrent infectious disease in over half (8/15) of the *C. psittaci*-positive cases examined microscopically. Avian pox was confirmed (using PCR +/- histology +/- electron microscopy) in five *C. psittaci-*positive great tits from separate incidents, including one (Case 16) with confirmed chlamydiosis (Table 4; Supplementary Table 2). Trichomonosis was diagnosed (using PCR and histology) in one *C. psittaci-*positive dunnock (Case 3) from a mortality incident affecting predominantly finch species (Table 3). Concurrent trauma, most commonly cat predation, was either confirmed or suspected in nine *C. psittaci-*positive cases, including four cases of confirmed chlamydiosis (Table 4).

## Discussion

When garden bird carcasses from 38 mortality incidents suggestive of chlamydiosis were examined retrospectively, chlamydiosis was diagnosed in at least one bird from each of nine incidents. Ten birds, submitted from 2006–2010, were positive for the disease: eight small passerines (5 dunnocks, 3 great tits) and two collared doves. The eight passerines were from seven separate mortality incidents, which add to eight previously confirmed incidents associated with chlamydiosis in small passerines in Britain (Simpson and Bevan 1989; Pennycott et al. 2009; Colvile et al. 2012). Colvile et al. (2012) described six small passerine chlamydiosis mortality incidents in England, five of which occurred in 2011 and questioned whether there had been a recent increase in the incidence of chlamydiosis in small passerines in Britain. Here, cases of passerine chlamydiosis were identified in each year of the study, indicating that any apparent increase in incidence is most likely to have been due to increased diagnostic effort. Furthermore, our results show that chlamydiosis is likely to have been a commoner cause of disease in small passerines than was previously recognized.

In addition to the 10 garden birds diagnosed with chlamydiosis, a further 11 birds found dead from 2005–2011 were positive for *C. psittaci* infection. Post-mortem tissue decomposition precluded histological or immunohistochemical examination in six of these cases, while in five cases there was equivocal evidence of chlamydiosis (tissues from four cases were negative on immunohistochemistry; one case had immunolabeling but no evidence of histological lesions consistent with chlamydiosis). Seven of the cases in which chlamydiosis was not confirmed (Cases 1, 3, 6, 13, 14, 18, and 19) had a high average Ct-value (38 or over) in the Chlamydiaceae-specific PCR assay, and in five of these cases at least one of the follow-up *C. psittaci-*specific assays was also negative, indicative of very low tissue concentrations of the bacterium. *C. psittaci* infection may not, therefore, have been a primary factor in the death of these birds and may have been incidental in some cases.

There was concurrent infectious disease in over half (8/15) of the *C. psittaci*-positive cases examined histologically: chlamydiosis was confirmed in 3 of these cases, while in 5 cases, histology was equivocal for chlamydiosis—indicating that another infectious disease may have been the primary cause of morbidity or death. Avian pox in great tits—an emerging infectious disease in Britain (Lawson et al. 2012)—was the most common concurrent infectious disease diagnosed (5 cases). In addition to chlamydiosis, a dunnock examined in the current study had trichomonosis. Concurrent chlamydiosis and trichomonosis were previously reported from a passerine mortality incident in Scotland in 2008 (Pennycott et al. 2009), and concurrent infectious disease is a common finding in other avian species affected by chlamydiosis (Vanrompay et al. 1995). At least four of the 21 *C. psittaci-*positive cases (including two cases with chlamydiosis) had evidence of cat predation. There have been rare reports of disease in cats and dogs associated with *C. psittaci* infection (Werth 1989), most commonly attributed to the animals having contact with pet parrots. The risk of pet cats or dogs acquiring the infection from wild birds is unknown but is likely to be low since there are few diagnosed cases of chlamydiosis in these companion animals.

Most (17 of 21) positive cases were selected for testing based on the presence of gross lesions typical of avian chlamydiosis (hepatomegaly, splenomegaly, and serositis), hence any *C. psittaci-*positive cases with different or no gross lesions would have been overlooked during case selection for this study. Also, only certain species were selected for diagnostic testing. It is therefore not possible to make inferences regarding the prevalence of chlamydiosis, or *C. psittaci* infection, in the general passerine population in Britain from this study. Further investigation, particularly of cases with no gross or macroscopic lesions (or clinical signs) typical of chlamydiosis, is warranted in order to explore the prevalence of *C. psittaci* infection in passerines.

Both the number and species of birds that had been observed sick or dead in each of the *C. psittaci-*positive mortality incidents we identified were highly variable. In the eight positive incidents in which there had been multi-species mortality, tits, dunnocks, robins, and finches were the most commonly affected species, as observed in previous studies (Simpson and Bevan 1989; Pennycott et al. 2009). Such a species complement, however, was one of the criteria used for the selection of cases for this study and was the sole basis for the selection of cases from three incidents, so this observation will be circular. No apparent sex predisposition to *C. psittaci* infection or seasonality of infection was evident, although the relatively small sample size may provide limited inferences regarding these factors. *C. psittaci*-positive incidents were widespread geographically (Fig. 1). Two *C. psittaci-*positive cases were from Wales, where (to the authors’ knowledge) infection with *C. psittaci* in free-living passerines has not been reported previously.

The use of four very sensitive assays—one family-specific screening assay, combined with three *C. psittaci*-specific assays—ensured that the overall molecular diagnostic method was highly sensitive and specific. *C. psittaci* was characterized as genotype A in all 15 passerines in which this could be determined. The sub-genotype was determined to be A-VS1 in 11 cases and A-6BC in 4 cases. A further six passerines diagnosed with chlamydiosis in a previous study (Colvile et al. 2012) also had genotype A-VS1. Genotype A has been identified most commonly in captive psittacines (Sachse et al. 2008, 2009), but our results suggest it is also a common genotype in wild passerines in Britain. Genotype A-VS1 is the most common subtype of genotype A, with the broadest host range of all *C. psittaci* genotypes, having previously been identified in psittacines, poultry, pigeons, canaries, and pheasants (Sachse and Rüttger 2014). Genotype A-6BC has been identified in a similar range of host species to A-VS1 but appears to be less prevalent (Sachse and Rüttger 2014). In two collared doves with chlamydiosis (Cases 10 and 15), *C. psittaci* was characterized as genotype E. Genotype E has been identified previously in feral pigeons (Magnino et al. 2009).

Although all *C. psittaci* genotypes are potentially zoonotic (Vanrompay et al. 2007), genotype A is the most commonly identified genotype in people, including in patients with severe psittacosis (Heddema et al. 2006; Vanrompay et al. 2007; Gaede et al. 2008). *C. psittaci* genotype A was identified in all four genotyped cases in a recent outbreak of human psittacosis in southern Sweden (that affected at least 25 people), in which wild passerines were implicated as the source of infection (Rehn et al. 2013). The identification of *C. psittaci* genotype A in passerines in the current study supports wild passerines as a potential source of human infection. Case-control investigations of human psittacosis outbreaks in Australia and Sweden have identified direct or indirect contact with live or dead wild birds (Telfer et al. 2005; Rehn et al. 2013), cleaning of wild bird feeders (Rehn et al. 2013), time spent in the garden, and lawn mowing (Williams et al. 1998; Telfer et al. 2005) as risk factors for disease. It is recommended that the public take sensible hygiene precautions when handling sick or dead wild birds and garden bird feeders, and that they wet areas contaminated with bird droppings prior to cleaning to minimize aerosolization, to reduce the risk of infection with *C. psittaci* and other zoonotic pathogens (Pennycott et al. 2009; Colvile et al. 2012; Rehn et al. 2013).

Although the overall risk of *C. psittaci* transmission from wild birds to humans is likely to be low (Haag-Wackernagel and Moch 2004; Rehn et al. 2013), considering that over 12 million households provide supplementary food for garden birds in Britain (Davies et al. 2009), it is important to determine the prevalence of subclinical *C. psittaci* carriage in wild passerines in order to understand the risks of zoonotic transmission (Colvile et al. 2012).

## Conclusion

Through this retrospective study, we almost double (from 8 to 15) the number of small passerine mortality incidents in Britain in which chlamydiosis has been diagnosed, showing that chlamydiosis may be a more common cause of disease in British passerines than was previously recognized. We diagnosed further cases of *C. psittaci* infection in passerines, and showed that it was unlikely to have been a primary pathogen in some birds. *C. psittaci* was characterized as genotype A in all the passerines (dunnocks, great tits, robins, and blue tits) from which it was determined, indicating that this is likely to be a common genotype in these species. As this genotype is known to be capable of infecting people, our results support a potential role for wild passerines in the zoonotic transmission of *C. psittaci*. Further research is required to determine the prevalence of *C. psittaci* infection in wild birds in Britain; people should be advised to take appropriate hygiene precautions when cleaning wild bird feeders or when handling sick or dead wild birds.

## Electronic supplementary material

Below is the link to the electronic supplementary material.

Details of mortality incidents and gross post-mortem examination findings in birds negative for *C. psittaci* infection. (DOC 56 kb)

*Chlamydia psittaci*-positive birds in which histology and immunohistochemistry were either not performed, or in which the results were equivocal for chlamydiosis. (DOC 53 kb)
