# Supplementary material for: Chlamydiosis in British Garden Birds (2005–2011): Retrospective Diagnosis and Chlamydia psittaci Genotype Determination
Source: Ecohealth. 2014 Jun 20;11(4):544–63. doi: 10.1007/s10393-014-0951-x (PMC4368850; doi:10.1007/s10393-014-0951-x)
Supplement: Supplementary file 1 — Details of mortality incidents and gross post-mortem examination findings in birds negative for C. psittaci infection. (DOC 56 kb) [file 10393_2014_951_MOESM1_ESM.doc]

| Case no. | Species and signalment | Details of mortality incident | | | Body condition, (bodyweight (g)) and gross findings on post mortem examination |
| --- | --- | --- | --- | --- | --- |
|  |  | Date and location | Species affected: no. found dead (no. seen sick) (*and total no. affected individuals*) | Clinical signs (if sick birds were observed) and/or perceived cause of death (reported by members of the public) |  |
| 22 | Robin  Adult | Jul 2006  Pembrokeshire, Wales | Robin 1 (0) | Possible window strike | Normal (17.0)  Skull fracture. Splenomegaly |
| 23 | Robin  Adult female | Jan 2007  Hampshire, England | Robin 1 (0) | None reported | Normal (19.3)  Skull fracture and haemorrhage. Pulmonary congestion. Suspected hepatomegaly |
| 24 | Wren  Juvenile | Jul 2007  Pembrokeshire, Wales | Wren 1 (0) | Possible window strike | Normal (9.3)  Suspected splenomegaly. Anorexia |
| 25 | Robin  Adult male | Jul 2007  Nottinghamshire, England | Robin 1 (1) (*1 individual*) | Possibly ‘sick’ before death | Thin (13.8)  Suspected splenomegaly. Anorexia |
| 26 | Chaffinch  Juvenile | May–Sep 2007  Devon, England | Greenfinch 25-30 (*some*)  Chaffinch 10-12 (*some*)  Collared doves 5 (*some*) | Some finches were seen gaping and/or with food on their beaks. Some finches were euthanized. Two collared doves were predated by a sparrowhawk | Thin (19.5)  Skull fracture. Necrotic ingluvitis (trichomonosis subsequently confirmed on further testing). Hepatomegaly |
| 27 | Collared dove  Adult female | Sep 2007  Greater London, England | Collared dove 1 (1) (*1 individual*) | Seen distressed prior to death | Normal (180)  Predator wounds. Fibrinous serositis |
| 28 | Rook  Adult male | Jul 2007–Sep 2008  Cheshire, England | Carrion crow 4-5 (*some*)  Rook 1 (1) (*1 individual*)  Jackdaw 1 (0) | Sick crows appeared to have ‘eye problems’ and were lethargic, as was the rook.  Jackdaw was found dead | Emaciated (345)  Air sacculitis, pneumonia and pericarditis |
| 29 | Jackdaw  Adult | From the same mortality incident as Case 28 (see above) | | | Normal (252)  Hepatic congestion and suspected hepatomegaly |
| 30 | Feral pigeon  Adult male | Aug 2008  Greater London, England | Feral pigeon 1 (0) | None reported | Thin (149)  Excess coelomic fluid? Anorexia |
| 31 | Jackdaw  Adult | Jun 2009  Somerset, England | Jackdaw 1 (0) | Suspected cat predation | Thin (163)  Wounds, fractures and haemorrhage. Hepatomegaly and suspected splenomegaly |
| 32 | Great tit  Juvenile | Jun 2009  Gwent, Wales | Great tit 1 (0) | Window strike | Normal (18.6)  Haemorrhage. Splenomegaly and suspected hepatomegaly |
| 33 | Feral pigeon  Juvenile | Aug 2009  Greater London, England | Feral pigeon 1 (1) (*1 individual*) | Found with ‘limp’ wing and euthanized | Thin (233)  Fractures, wound and haemorrhage. Serositis. Suspected hepatomegaly |
| 34 | Rook  Adult | Jul – Sep 2009  Bedfordshire, England | Rook 21 (*some*)  Bullfinch 0 (1) | Most rooks found dead. Sick birds generally appeared weak, struggling to walk and fly | Emaciated (274)  Pneumonia and air sacculitis. Suspected hepatomegaly |
| 35 | Pied wagtail  Adult male | Jan 2010  Suffolk, England | Pied wagtail 1 (1) (*1 individual*) | Fluffed up and lethargic before death | Emaciated (16.5)  Serositis. Suspected hepatomegaly |
| 36 | Great tit  Nestling | May 2010  Derbyshire, England | Great tit 5 (0)  Blue tit 1 (0) | All of a clutch of 5 nestlings found dead.  Single nestling found dead | Normal (6.1)  Diffuse pulmonary congestion |
| 37 | Great tit  Nestling | May 2010  Essex, England | Blue tit 3 (0)  Great tit 3 (0)  Blackbird 1 (0) | All of 3 nestlings in 1 clutch of blue tits and 1 clutch of great tits died  None reported | Normal (10.5)  Suspected wound and possible intestinal lesion |
| 38 | Great tit  Adult | Nov 2011  Cambridgeshire, England | Great tit 1 (1) (*1 individual*) | Cat predation, skin ‘lump’ | Thin (18.0)  Large skin lesion (avian pox subsequently confirmed on further testing). Probable skull fracture. Splenomegaly |
| 39 | Great tit  Adult | Oct 2010  Derbyshire, England | Great tit 1 (0) | Suspected predation | Normal (17.6)  Fractures and haemorrhage. Suspected splenomegaly |
| 40 | Blue tit  Adult male | Mar 2011  Dorset, England | Blue tit 1 (1) (*1 individual*) | Fluffed up and lethargic before death | Thin (8.7)  Anorexia |
