# Supplementary material for: Chlamydiosis in British Garden Birds (2005–2011): Retrospective Diagnosis and Chlamydia psittaci Genotype Determination
Source: Ecohealth. 2014 Jun 20;11(4):544–63. doi: 10.1007/s10393-014-0951-x (PMC4368850; doi:10.1007/s10393-014-0951-x)
Supplement: Supplementary file 2 — Chlamydia psittaci-positive birds in which histology and immunohistochemistry were either not performed, or in which the results were equivocal for chlamydiosis. (DOC 53 kb) [file 10393_2014_951_MOESM2_ESM.doc]

| Case no. | Species and signalment | Results of microbiological examination and additional tests | Histopathological findings | Immunohistochemical labelling for *Chlamydia* sp. specific antigens | Diagnoses |
| --- | --- | --- | --- | --- | --- |
| 1 | Blue tit  (Adult) | Liver: predominantly *Enteroccus* sp.. Lung: *Escherichia coli* 1. Small intestine (SI): no growth | Not done^b^ | Not done^b^ | *C. psittaci* infection |
| 3 | Dunnock  (Adult) | Liver, SI and crop lesion: predominantly *E. coli* 1. Crop: also *Enterococcus* sp..  Crop lesion: *Trichomonas* sp.-specific PCR positive | Marked, fibrinonecrotic to granulomatous ingluvitis compatible with *Trichomonas* sp. infection, and probable associated Gram-negative bacterial infection. Hepatic microthrombosis. Giemsa, Ziehl-Neelsen (ZN) and Periodic Acid-Schiff (PAS) stains negative for inclusion bodies | Brain, trachea, liver, heart, crop and pectoral muscle all devoid of immunolabelling | Trichomonosis;  probable associated bacterial infection;  *C. psittaci* infection |
| 6 | Great tit  (Adult) | SI: few colonies *E. coli* 1 and *Enterococcus* sp.. Lung: non-haemolytic *Staphylococcus* sp.. Skin: non-haemolytic *Staphylococcus* sp. and *Citrobacter freundii*.  Avian pox infection confirmed on avipox PCR and electron microscopy | Proliferative, necrotizing, extensive dermatitis with numerous intracytoplasmic inclusion bodies (pathognomonic for avian poxvirus infection) and intralesional bacterial cocci (compatible with the *Staphylococcus* spp.). Pectoral muscle: focal, marked, vascular endothelial hypertrophy, with cytoplasmic bodies (intracellular bacteria). Fibrinous, locally extensive, acute, pneumonia with atelectasis and congestion. Fibrinous splenitis. Hepatic perivascular cellular infiltrates (interpretation hindered by autolysis). Blood cells appear to contain cytoplasmic bodies resembling a hemoparasite such as *Leucocytozoon* spp.. Acute pectoral muscle haemorrhage. ZN and Giemsa stain reveal no Chlamydial inclusion bodies | Brain, trachea, heart, liver, lung, spleen, pectoral muscle and skin lesions devoid of immunolabelling | Avian pox disease with secondary infection by *Staphyloccus* sp.;  *C. psittaci* infection and disseminated bacterial infection (possible chlamydiosis);  probable protozoan hemoparasitism;  cat predation |
| 7 | Great tit  (Adult) | SI: *E. coli* 1 and *Enterococcus* spp.. Lung: *Enterococcus* sp. and β-haemolytic *S. aureus*. Skin: non-haemolytic *Staphylococcus* sp. Yeast sp. (other than *C.albicans*) and *Serratia marcescens*.  Skin lesion: avipox PCR positive | Proliferative, necrotizing, extensive dermatitis with numerous intracytoplasmic inclusion bodies (pathognomonic for avian poxvirus infection), with heavy secondary infection by intralesional bacterial cocci (compatible with the *Staphylococcus* spp.). No lesions of chlamydiosis visible in liver or lung, but interpretation hindered by severe autolysis. ZN and Giemsa stains reveal no Chlamydial inclusion bodies | Brain, trachea, heart, liver, lung, pectoral muscle and skin lesions were devoid of immunolabelling | Avian pox disease with secondary *Staphylococcus* sp. infection/  septicaemia;  euthanasia;  *C. psittaci* infection |
| 12 | Dunnock  (Adult male) | Liver and lung: Light growth *E. coli* 1 and *Pseudomonas fluorescens*. SI: Mixed non-haemolytic *Staphylococcus* sp., *Enterococcus* sp, *P. fluorescens* and *P. aeruginosa* | Not done^b^ | Not done^b^ | Possible trauma;  *C. psittaci* infection |
| 13 | Robin  (Nestling) | Liver: Mixed *E. coli* 1 and *Hafnia alvei*. SI: *H. alvei* and *C. freundii* | Not done^b^ | Not done^b^ | *C. psittaci* infection |
| 14 | Blue tit  (Nestling) | Liver: *E. coli* 1 and *Enterococcus* spp.. Lung: mixed, predominantly *E. coli* 1. No *Suttonella* sp.. SI: no growth | Not done^b^ | Not done^b^ | *C. psittaci* infection |
| 18 | Great tit  (Adult female) | Liver, SI and lung: *Enterococcus* sp. and *S. aureus*.  Skin lesion: avipox PCR positive | Proliferative, necrotizing, severe dermatitis with numerous intracytoplasmic inclusion bodies (pathognomonic for avian poxvirus infection) and intralesional bacterial cocci (consistent with *Staphylococcus* sp.). Focal encysted protozoan megaloschizont in intestine. Liver: possible intraerythrocytic or intraleucocytic protozoan life stages. Trachea: intraluminal metazoan (nematode) parasite. Severe adipose atrophy. ZN stain highlights oval structures (approximately 20 μm diameter) in the proventricular lumen (insect parts or plant material). ZN and Giemsa stains: no inclusion bodies | Positive labelling in the  tracheal mucosa, liver and pox lesion. (Brain, heart, pectoral muscle, lung and proventriculus and gizzard devoid of immunolabelling) | Avian pox disease and secondary *S. aureus* septicaemia;  hemoparasitic infection (probable *Leucocytozoon* sp.);  possible gapeworm or similar tracheal parasitic infection;  *C. psittaci* infection (possible chlamydiosis) |
| 19 | Dunnock  (Adult male) | Liver: Pure isolate *P. fluorescens*. SI: *P. fluorescens* and *E. coli* 1 | Not done^b^ | Not done^b^ | Euthanasia;  *C. psittaci* infection |
| 20 | Great tit  (Adult female) | Liver and skin lesion: pure isolate β-haemolytic *S. aureus*. SI: *E. coli* 1.  Skin lesion: avipox PCR positive | Not done^b^ | Not done^b^ | Avian pox disease and secondary *S. aureus* septicaemia;  cat predation;  *C. psittaci* infection |
| 21 | Blue tit  (Adult male) | Liver, SI and lung: pure isolate *E. coli* 1.  Tissues negative for *Suttonella* sp. (on culture) and *Mycoplasma* sp. (on PCR)^a^ | Bilateral, fibrinous, extensive, exudative conjunctivitis with intralesional bacteria; many bacterial coccobacilli or rods within the conjunctival exudate. Mild pustular blepharitis. Cataract (aetiology uncertain). Extensive pulmonary congestion, oedema and atelectasis with circulating bacteria (similar morphology to those in the conjunctival exudate). ZN, PAS and Giemsa stains highlight bacteria in the conjunctival exudate and reveal no other agents or inclusion bodies | Eye, skull, testis, brain, liver, heart, proventriculus and gizzard, pectoral muscle, lung, trachea and oesophagus all devoid of immunolabelling | Apparent disseminated bacterial infection, possibly originating from severe conjunctivitis;  *C. psittaci* infection |

^a^*Mycoplasma* sp. PCR performed by the University of Liverpool, Leahurst, England, CH64 7TE, UK

^b^Histopathological and immunohistochemical examinations were not performed in these cases owing to tissue decomposition
